# *Drosophila* innate immunity suppresses the survival of xenografted mammalian tumor cells

**DOI:** 10.1038/s41598-023-38489-9

**Published:** 2023-07-30

**Authors:** Ayaka Aida, Kevin Yuswan, Yoichi Kawai, Keita Hasegawa, Yu-ichiro Nakajima, Erina Kuranaga

**Affiliations:** 1 Laboratory for Histogenetic Dynamics, Graduate School of Life Sciences, Tohoku University, Sendai, 980-8578 Japan; 2 Frontier Research Institute for Interdisciplinary Sciences, Tohoku University, Sendai, 980-8578 Japan; 3 Graduate School of Pharmaceutical Sciences, The University of Tokyo, Tokyo, 113-0033 Japan; 4 Laboratory for Histogenetic Dynamics, Graduate School and Faculty of Pharmaceutical Sciences, Kyoto University, Kyoto, 606-8304 Japan

**Keywords:** Cancer, Cell biology, Diseases

## Abstract

Patient-derived xenograft (PDX) is an emerging tool established in immunodeficient vertebrate models to assess individualized treatments for cancer patients. Current xenograft models are deficient in adaptive immune systems. However, the precise role of the innate immunity in the xenograft models is unknown. With conserved signaling pathways and established genetic tools, *Drosophila* has contributed to the understanding of the mechanism of tumor growth as well as tumor–host interactions for decades, making it a promising candidate model for studying whether or not the hosts’ innate immunity can accommodate transplanted human tumor cells. Here we show initial observations that assess the behavior and impact of several human tumor cell lines when transplanted into *Drosophila*. We found that some injected cell lines persisted for a longer duration and reduced hosts’ lifespan. In particular, the human lung cancer cell line A549 were observed adjacent to the fly host tissues. We examined two factors that affect the survivability of cancer cells: (1) the optimal temperature of each cell line and (2) the innate immunity of *Drosophila* hosts. Especially, transplanted human tumor cells survived longer in immunodeficient flies, suggesting that the host innate immune system impedes the growth of xenografted cells. Our attempts for xenografting fly models thus provide necessary steps to overcome for establishing PDX cancer models using invertebrates.

## Introduction

Non-clinical tests are mandatory in the strict development process of cancer therapeutics to ensure drug safety and efficacy prior to human trials. Mouse models have been the most commonly used animal model for non-clinical tests among which the patient-derived xenograft (PDX) model has proven to be an emerging technique^1^. In the PDX model, a patient’s tumor tissue is directly transplanted into immunodeficient mice to mimic the patient’s cancer^2^, and then drug tests are conducted to identify the most effective treatments against the case, as personalized medicine. Despite their established protocols, mouse-based xenograft models are expensive and time-consuming to generate, requiring specialized pathogen-free facilities, and the ethical limitations prevent large-scale screening. Therefore, establishing a simpler drug screening model that can lead to a breakthrough in the development of personalized medicine is desired.

The fruit fly *Drosophila melanogaster* has been used as a model for understanding mechanisms of human diseases including cancers due to the high number of conserved genes and molecular pathways with approximately 75% of human disease-related genes having *Drosophila* homologs^3,4^. Together with the intrinsic characteristics represented by short lifespan and high reproduction rate, these features allow researchers to perform large-scale research and screening at lower costs compared to vertebrate models. Accordingly, *Drosophila* has been used for studying different aspects of tumor growth for many years^3,5,6^. Especially, following the development of microinjection techniques by Beadle and Ephrussi in 1935^7^, allograft tumor assays with injections of larval tissues into adult hosts have proven quite useful for investigating mechanisms of tumor–host interactions^8^. One example of a successful allograft model is the *Ras*^*V12*^*/scrib*^*−/−*^ tumors derived from eye-antennal discs^9^ wherein expression of the mutant oncogene *Ras*^*V12*^ and loss of the neoplastic tumor suppressor gene *scribble* (*scrib*). Following allograft transplantation into adult flies, the *Ras*^*V12*^*/scrib*^*−/−*^ tumor tissues exhibit extensive proliferation and invasion into the neighboring tissues^9–11^.

Despite many fruitful achievements in allograft models, however, xenografting experiments in *Drosophila* have not been established. This may be due to the extreme differences in the body structure, organ systems, and living conditions between vertebrates and *Drosophila*^5^*.* One factor that may impact the survival and growth of xenografted cells is the immune system of the hosts. Compared to vertebrates that have both acquired and innate immunity, the *Drosophila* immune system only consists of the innate immunity, which is regulated by the Toll and Imd pathways^12^. Various studies have shown the immune system’s efficacy in removing bacteria and fungi through the production of antibacterial peptides, such as Drosomycin and Diptericin^13^. However, whether or not the innate immune system can protect the hosts from foreign cells derived from nonbacterial and nonfungal origins is unclear.

In this study, we performed xenograft experiments by injecting human tumor cell lines into adult *Drosophila* hosts. Among cell lines that we injected, A549 and DLD-1 cells survived for approximately 12 days within the control hosts, and these host flies exhibited significantly shortened life span. We further show that the human tumor cells remained longer and spread more widely when transplanted into immunodeficient host flies, which causes mortality of host flies. These results together suggest that the *Drosophila* innate immune system plays a role in negatively influencing human tumor cell growth, providing a caution for the influence of the innate immune system when generating PDX models.

## Materials and methods

### *Drosophila* culture and stocks

*y*, *w*, *ey-Flp; Act*>*y*+>*Gal4*, *UAS-GFP; FRT82B*, *Tub-Gal80* and *UAS-Ras*^*V12*^ were a gift from T. Xu (Yale University School of Medicine). *FRT82B*, *scrib*^*1*^*/TM6B*^14^ was a gift from D. Bilder (University of California, Berkeley). *Rel*^*E20*^ (stock number 55714), *Dredd*^*D44*^ (stock number 80924), and *PGRP-LC*^*Δ5*^ (stock number 36323) were obtained from the Bloomington *Drosophila* Stock Center (Indiana, USA). *w*^*1118*^ was maintained at 25 °C on standard medium. *Rel*^*E20*^, *Dredd*^*D44*^, and *PGRP-LC*^*Δ5*^ were maintained at 20 °C on standard medium.

### Cell culture

The mammalian cell lines were maintained at 37 °C, 5% CO_2_ in Dulbecco’s modified Eagle medium (DMEM; high glucose) purchased from Wako (Japan, Catalog No. 044-29765), supplemented with 10% fetal bovine serum (FBS; Biosera, Nuaille, France, Catalog No. 12389) and 100 µm/ml penicillin and 100 μg/ml streptomycin (Nacalai Tesque, Kyoto, Japan, Catalog No. L5B3459).

HeLa Kyoto EGFP-alpha-tubulin/H2B-mCherry, A549 YFP-keratin8, MCF7 YFP-keratin8, and DLD-1 GFP-lifeact were gifts from K. Ohashi (Tohoku University, Sendai, Japan).

### Generation of *Ras*^*V12*^*/scrib*^*−/−*^ clones and allograft protocol

Fluorescent-labeled clones were generated in larval eye discs using the ey-FLP MARCM (eyMARCM) system^9^. Following dissection and collection of GFP (Green Fluorescent Protein)-labeled *Ras*^*V12*^*/scrib*^*−/−*^ malignant tumor tissue from third-instar larvae, the tumors were treated with 0.083% trypsin (Wako, Japan, Catalog No. TWF7014) for 5 min, passed through a 27-G needle (TERUMO, Japan, Catalog No. NN-2719S) with a 1-ml syringe, and finally suspended in phosphate-buffered saline (PBS; NaCl, KCl, Na_2_HPO_4_, KH_2_PO_4_; Wako).

The cell suspension was extracted into a glass needle (Drummond Scientific Company, Broomall, Pennsylvania, USA, Catalog No. 1-000-0300) and then injected into the abdomen of young adult female flies (within 5 days upon eclosion) using a NARISHIGE IM300 Microinjector (Narishige Scientific Instrument Lab., Tokyo, Japan) with the following settings: N_2_ gas fill pressure at 20.0 PSI, injection pressure at 5.0 PSI, and balance pressure at 1.8 PSI. Injected host flies were incubated at 29 °C.

### Human tumor cell line injection

The human tumor cell lines, HeLa Kyoto EGFP-alpha-tubulin, A549/YFP-keratin8, MCF7/YFP-keratin8, and DLD-1/GFP-lifeact were trypsinized with 0.05–0.083% trypsin, then pelleted and resuspended in 1 ml PBS for cell counting. After counting, cells were transferred to a new PBS suspension at the desired concentration.

**Figure 1:**
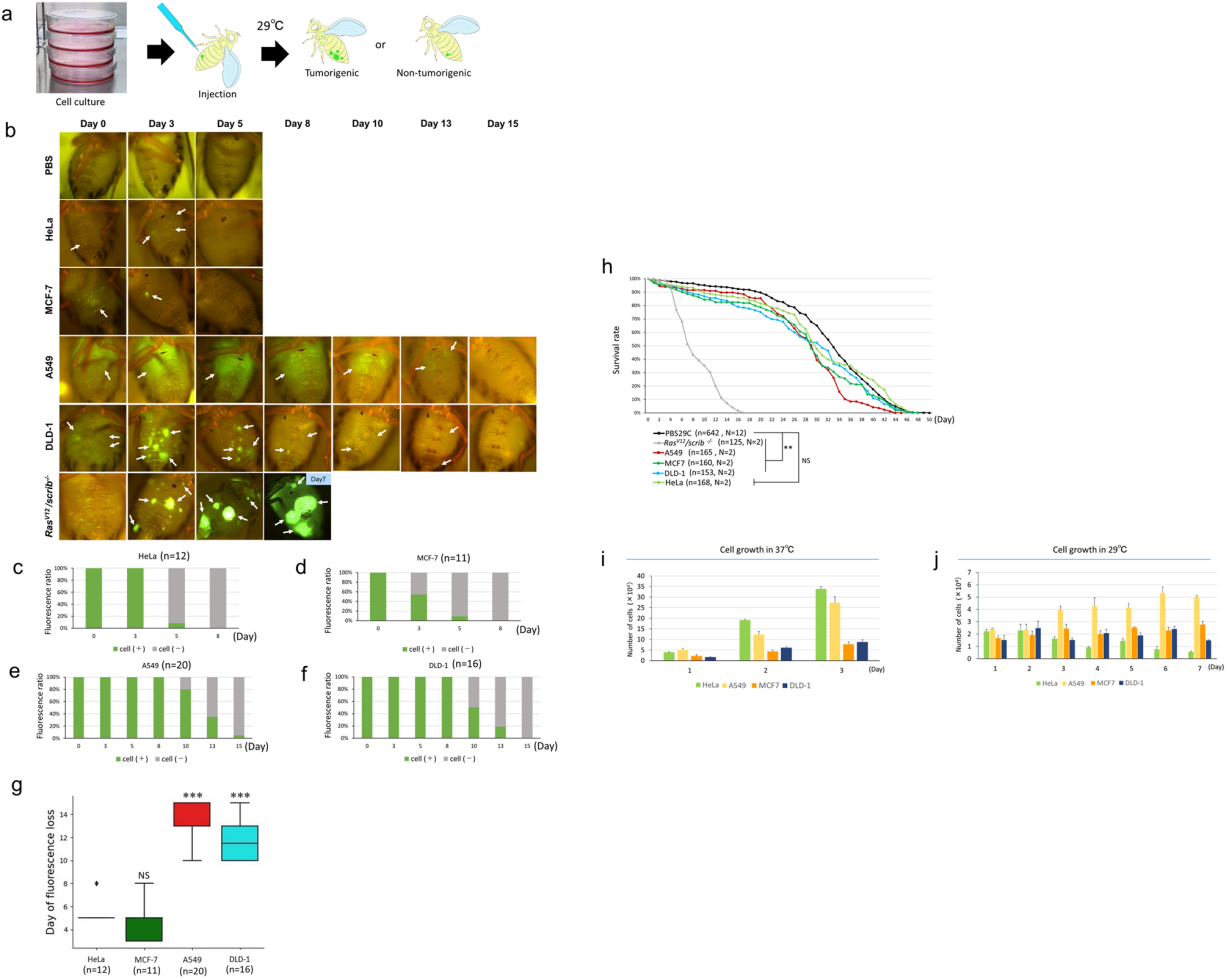
Injected human tumor cells remain in fly hosts and affect hosts’ lifespan. (**a**) Experimental scheme of human tumor cell line injection. The abdomen of young adult female flies (within 5 days after eclosion) are injected with cultured human tumor cell lines in suspension. During incubation at 29 °C, we obtained survival rate and observed the fluorescence of tumor cell lines in the abdomen through fluorescence microscopy, to evaluate tumor spread. (**b**) Fluorescence microscopy images of the female abdomen injected with PBS, human tumor cells (HeLa, MCF-7, A549, and DLD-1), or *Drosophila Ras*^*V12*^*/scrib*^*−/−*^ cells over time. Arrows indicate the tumor locations. PBS injection is the negative control. Cell concentration was 600 × 10^4^ cells/50 μl PBS for human cell injection and 300 × 10^4^ cells/50 μl for *Drosophila Ras*^*V12*^*/scrib*^*−/−*^ cells injection. (**c**–**f**) Ratio of individuals with observable tumor cell fluorescence on day 0, 3, 5, 8, 10, 13 and 15. (**g**) Boxplot of the day when host flies clear the indicated cells by fluorescence loss. Student’s *t*-test vs HeLa; *NS* not significant (P > 0.05), ***P < 0.001. (**h**) Survival rate of individuals injected with various types of cells (human tumor cells or *Drosophila* cells) or PBS. Log-rank test; *NS* not significant (P > 0.05). P-values are as follows: **P < 0.01. (**i**,**j**) Human tumor cell growth in culture at 37 °C (**i**) and 29 °C (**j**). Genotype of host flies: *w*^1118^.

**Figure 2:**
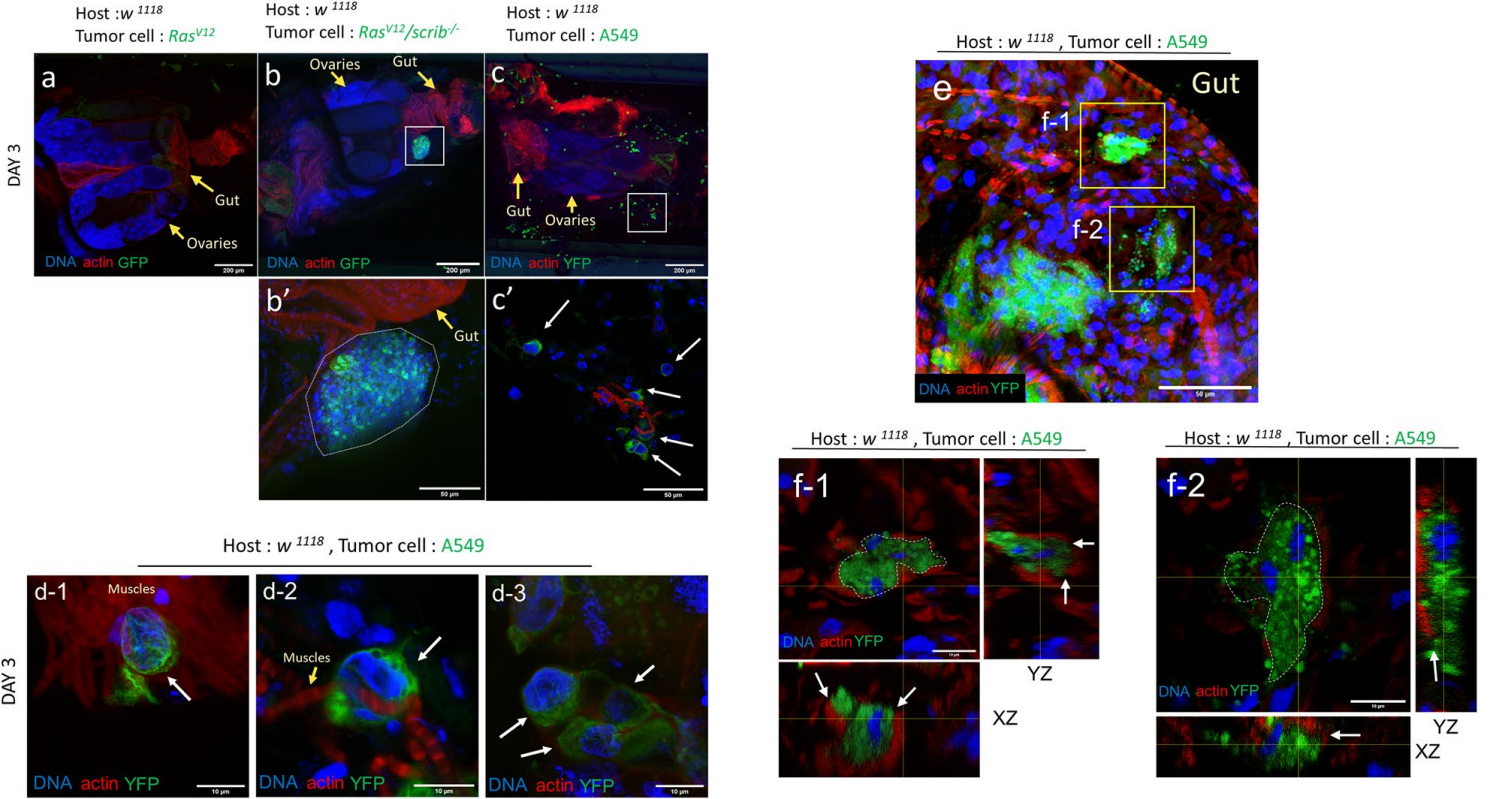
A549, human lung cancer cells are adjacent to host tissues. (**a**–**c**) Representative confocal laser microscopy images of the fly abdomen injected with the *Ras*^*V12*^ benign tumor cells (**a**), *Ras*^*V12*^*/scrib*^*−/−*^ malignant tumor cells (**b**), and A549 cells (**c**) on day 3 (*Ras*^*V12*^; n = 28, *Ras*^*V12*^*/scrib*^*−/−*^; n = 35, A549; n = 26). Yellow arrows indicate the specified tissues or organs. (**b**′) *Ras*^*V12*^*/scrib*^*−/−*^ tumor cluster (n = 7/35). Dotted lines surround the cell clusters. Yellow arrows indicate the specified tissues or organs. (**c**′) A549 cells (n = 15/26) in fly abdomen. White arrows indicate A549 cells. (**d**) A549 cells are adjacent to muscles (**d-1**,**d-2**). Some cells are adjacent to each other and formed small clusters (**d-3**). White arrows indicate A549 cells. Yellow arrows indicate the specified tissues or organs. (**e**) A549 cell clusters in the gut. Yellow boxed areas indicate the tumor clusters shown in (**f-1**) and (**f-2**). (**f**) Orthogonal views of A549 cells clusters in the smooth muscle layer in the gut shown in (**e**). Dotted lines surround the cell clusters. White arrows indicate the possible contact between A549 cells and host tissues. Scale bars: (**a**–**c**): 200 μm; (**b**′,**c**′,**e**): 50 μm; (**d**,**f**): 10 μm. Genotypes: (**a**) Host: *w*^1118^, Tumor donor: *y*,*w, ey-Flp; Act*>*y*+>*Gal4*,*UAS-GFP/UAS-Ras*^*V12*^*; FRT82B,Tub-Gal80/FRT82B*. (**b**,**b**′) Host: *w*^1118^, Tumor donor: *y,w, ey-Flp; Act*>*y*+>*Gal4,UAS-GFP/UAS-Ras*^*V12*^*; FRT82B,Tub-Gal80/FRT82B, scrib*^*1*^. (**c**–**f**) Host: *w*^1118^, Tumor donor: A549 cells.

**Figure 3:**
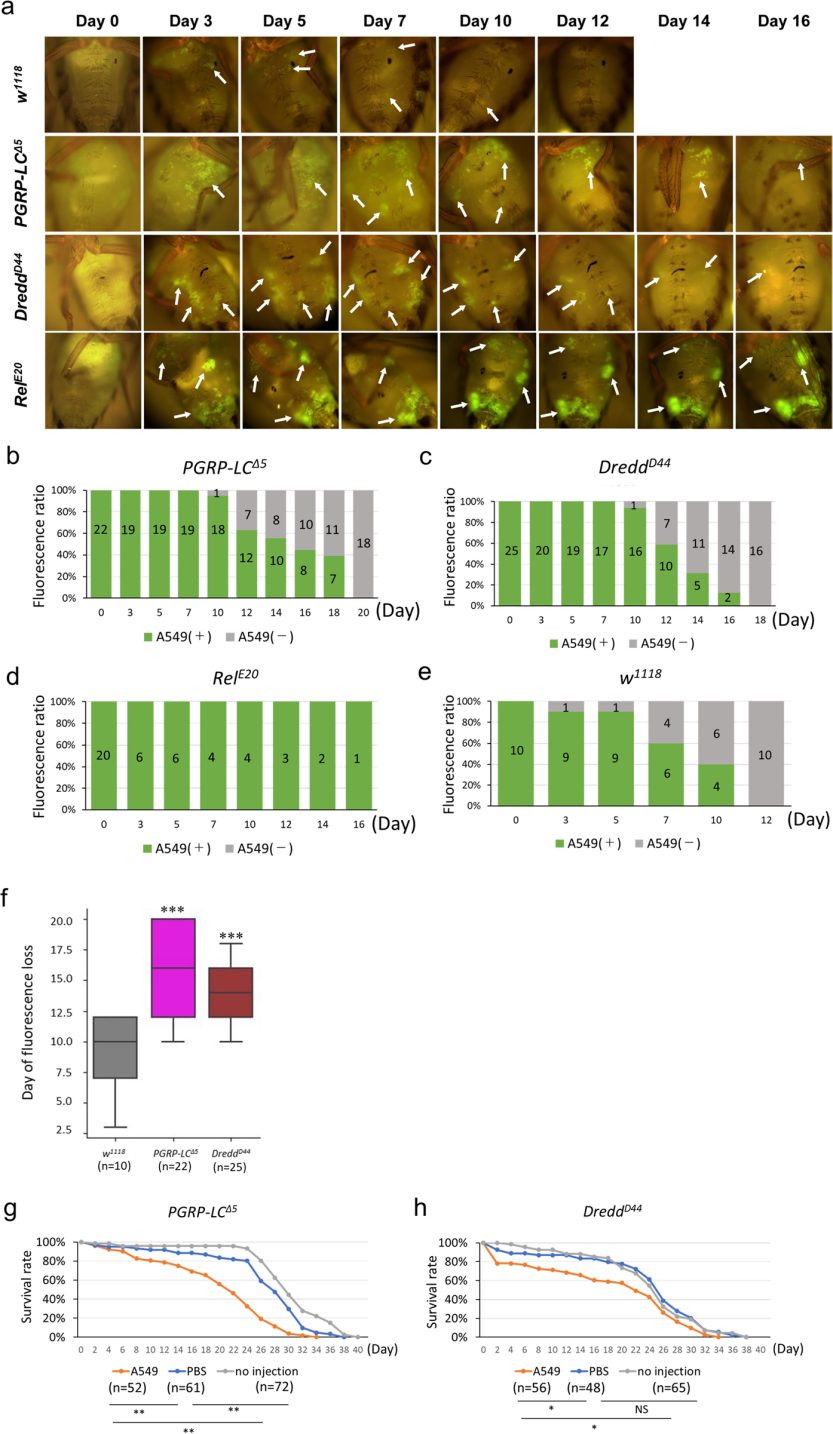
*Drosophila* innate immunity affects the transplanted human tumor cell survival. (**a**) Fluorescence microscopy images of *w*^*1118*^, *PGRP-LC*^*Δ5*^, *Dredd*^*D44*^, *Rel*^*E20*^ fly abdomen injected with A549 cells on day 0, 3, 5, 7, 10, 12, 14, and 16. Arrows indicate the location of tumors. Cell concentration was 300 × 10^4^ cells/50 μl PBS. (**b**–**e**) Ratio of individuals with observable tumor cell fluorescence on the specified days. Numbers on the bars represent the number of flies with or without tumor cell fluorescence at each time point. (**f**) Boxplot of the day when host flies clear the A549 cells by fluorescence loss. Note that *Rel*^*E20*^ is not included due to the death of hosts before clearing the A549 cells. Student’s *t*-test vs *w*^*1118*^, ***P < 0.001. (**g**,**h**) Survival rate of A549 cell injected *PGRP-LC*^*Δ5*^ (**g**) or *Dredd*^*D44*^ (**h**) hosts, compared to PBS injected control hosts and non-injected hosts. Log-rank test; *NS* not significant (P > 0.05). P-values are as follows: *P < 0.05, **P < 0.01. Genotypes: Hosts: *w*^1118^*, PGRP-LC*^*Δ5*^*, Dredd*^*D44*^*, Rel*^*E20*^.

The cell concentrations for injections were (1) 600 × 10^4^ cells/50 μl PBS for the human tumor cells and 300 × 10^4^ cells/50 μl PBS for the *Ras*^*V12*^*/scrib*^*−/−*^ injections in Figs. 1, 2, Fig. S1, and (2) 300 × 10^4^ cells/50 μl PBS for the A549 cells injection into immunodeficient flies in Fig. 3.

The cell suspension was injected into the young adult female abdomen as specified in the allograft protocol above. Fly hosts were *w*^*1118*^, *Rel*^*E20*^, *Dredd*^*D44*^, or *PGRP-LC*^*Δ5*^ as specified in the corresponding experiments in the result section. Injected host flies were incubated at 29 °C.

### Temperature-controlled cell growth assay

The human tumor cell lines, HeLa Kyoto EGFP-alpha-tubulin/H2B-mCherry, A549/YFP-keratin8, MCF7/YFP-keratin8, and DLD-1/GFP-lifeact were trypsinized with 0.05–0.083% trypsin and then pelleted and resuspended in 1 ml PBS. Cells were counted on a Burker-Turk Counting chamber (Sunlead Glass Corporation, Saitama, Japan) and seeded at 5.0 × 10^4^/well in 6-well plates. Cells were then incubated at 37 °C for 3 days or 29 °C for 7 days while being quantified every day.

### *Drosophila* life-span measurements

Flies were transferred to fresh food every day for 7 days following injection to avoid infection and every 2 days thereafter. Living flies were counted every 2 days. Lifespan analyses were performed, and log rank test statistics were calculated using the R version 4.0.2 (The R foundation, 2020).

### Histochemistry

At 3 days following injection, host fly abdomens were dissected, and the ventral epidermis were removed. Dissected abdomens were washed with PBS to remove floating cells in the hemolymph, leaving only the cells that had interacted with host tissues. Samples were fixed in 4% paraformaldehyde (PFA) and then washed with PBS 3 times. Cell nuclei were stained with Hoechst 33342 (Life Technologies, USA, Catalog No. H1399), and F-actin was stained with Alexa 555 Phalloidin (1:500; Life Technologies, Catalog No. A34055). The samples were washed with 0.1% TritonX-100/PBS (PBST) and then mounted on slide glass.

All imaging was conducted using a Leica TCS SP8 confocal laser scanning microscope with either a 40 × or 63 × NA1.3 oil-immersion objective (Leica Microsystems, Wetzlar, Germany). Image analyses were conducted using the ImageJ 1.53c (National Institutes of Health, USA, URL: https://www.google.com/search?q=http%3A%2F%2Fimagej.nih.gov%2FijJava+1.8.0%255F202(64-bit)) (64-bit)).

## Results

### Injected human tumor cells remain in fly hosts and affect hosts’ lifespan

Ectopic transplantation of tumorigenic tissues and cells such as *Ras*^*V12*^*/scrib*^*−/−*^-expressing eye discs and *Ras*^*V12*^*-*expressing primary cell lines into adult flies have been reported to undergo over-proliferation and promote the death of hosts^10,15,16^. To determine the behavior of human tumor cells in the fly body, we injected fluorescent-tagged cultured human tumor cells into flies’ abdomens using the established allograft protocol with modifications (see the “Materials and methods” for details)^8,10^. We used the following human tumor cell lines for xenografting: (1) HeLa-Kyoto (human cervical carcinoma cells), (2) A549 (lung epithelial adenocarcinoma cells), (3) MCF7 (breast cancer cells), and (4) DLD-1 (colorectal adenocarcinoma cells) (see “Materials and methods” for the specific cell names and culturing methods). After transplantations of human tumor cells into host flies, we monitored the fluorescence of tumor cells in the abdomen through fluorescence microscopy to evaluate tumor spreading and examined the survival rate of host flies (Fig. 1a), but no fluorescence was observed when PBS alone was injected (Fig. 1b [row 1]).

Throughout our observations, the fluorescence of HeLa cells and MCF-7 cells disappeared 3–5 days after injection (Fig. 1b [rows 2 and 3], c,d,g). In contrast, the fluorescence of A549 cells and DLD-1 cells persisted for 10–13 days (Fig. 1b [rows 4 and 5], e–g). In addition to the duration of fluorescent signals, we observed differences in the patterning of the tumor fluorescence: A549 cells spread and persisted in broader areas in the abdomen while DLD-1 cells were frequently clustered, exhibiting spotty fluorescence within the host abdomen (Fig. S1). As a positive control for tumorigenic proliferation in host flies, we injected *Ras*^*V12*^*/scrib*^*−/−*^ cells and observed the gradual spread of *Ras*^*V12*^*/scrib*^*−/−*^ cells over 7 days in the host abdomen, which was associated with extremely low survival rate (Fig. 1b [row 6], h). While xenograft flies with human tumor cells survived longer than the *Ras*^*V12*^*/scrib*^*−/−*^*-*transplanted flies, xenograft flies died significantly earlier than the PBS-injected control flies, except for those transplanted with HeLa cells (Fig. 1h).

Overall, human tumor cells were able to survive for several days in the host flies until they were cleared out (Fig. 1b–g). In contrast, *Drosophila Ras*^*V12*^*/scrib*^*−/−*^ cell-transplanted flies exhibited expansion of the fluorescence (Fig. 1b [row 6]). One potential factor influencing this decreased viability of human tumor cells in fly bodies was the optimal temperature difference between human cells and fly cells. The reported optimal temperature for healthy *Drosophila melanogaster* is between 14–29 °C, with a common incubation temperature of 25 °C in laboratories^17,18^. By contrast, mammalian cells are commonly cultured at a much higher temperature of 37 °C and likely experience cellular stress when cultured at lower temperatures^19^. We thus chose 29 °C for the incubation of fly hosts as an intermediate temperature between the optimum temperatures for flies and humans.

To test whether human tumor cell lines exhibit growth at this intermediate temperature, we cultured these cell lines in vitro at 29 °C. As expected, human tumor cell lines grew exponentially at different rates at 37 °C, with HeLa and A549 cells having the best growth rates (Fig. 1i). By contrast, we found a decreased growth rate of most tumor cell lines when incubated at 29 °C (Fig. 1j). While HeLa cells showed a decrease in numbers over time at 29 °C, A549 cells still maintained proliferation, albeit at a slower rate (Fig. 1j). These results suggest that different cell lines have distinct growth rates at 37 °C and 29 °C, indicating differences in their capabilities to adapt to lower temperatures.

Based on xenograft and in vitro culture experiments, we observed a correlation between the temperature resistance of the cell lines and their behavior following transplantation into fly hosts: (1) A549 cells, which continue to be sufficiently proliferative when cultured at 29 °C, showed persistence of GFP fluorescence up to around 13 days and induced a lower survival rate in host flies after transplantation; and (2) HeLa cells, which have lower resistance to low temperatures than the other cells, showed an inability to survive for longer than 3 days. Given that both HeLa and MCF-7 cells disappeared from *Drosophila* bodies within 5 days despite their continued survival at 29 °C in vitro, we considered a possibility that additional factors contribute to the removal of human tumor cells from fly hosts.

### Transplanted human lung cancer cells are clustered adjacent to fly host tissues

Our lifespan measurement results suggest that the presence and behavior of human tumor cells detrimentally affect the systems of host flies (Fig. 1b–h). To investigate the characteristics and potential interactions between human tumor cells and fly tissues at the cellular level, we observed the fluorescently labeled human tumor cells in detail in host flies at 3 days after transplantation.

Prior to fluorescent staining for host flies, we washed our samples with PBS to remove cells suspended in the hemolymph. Following fixation and staining, we imaged the samples using confocal laser microscopy. Of note, we chose A549 cells for the following observations due to their longer persistence in fly hosts than other cell lines. In addition to the A549 cells, we also performed allografts and observed two *Drosophila* tumor models—*Ras*^*V12*^ (Fig. 2a) and *Ras*^*V12*^*/scrib*^*−/−*^ (Fig. 2b,b′)—as negative and positive controls in terms of tumorigenicity, respectively^10^. As expected, *Ras*^*V12*^*/scrib*^*−/−*^ cells grew better and formed bigger clusters than *Ras*^*V12*^ benign tumor (Figs. 1b, 2b,b′).

After transplantation, we confirmed the presence of A549 cells within the host’s tissues sporadically located adjacent to fly host tissues (Fig. 2c,c′,d). In addition, some of the cells formed well-defined clusters within the muscle layer of the gut (Fig. 2e,f-1,f-2). These observations revealed that A549 cells, despite a different origin with a reduced proliferation rate at 29 °C compared to 37 °C, still displayed clustering behaviors, similar to *Ras*^*V12*^*/scrib*^*−/−*^ cells. Together with the lifespan measurements, these results suggest that other factors play roles in preventing the survival of A549 cells in host flies.

### *Drosophila* innate immunity suppresses transplanted human tumor cell survival

It is widely known that *Drosophila*, like many insects, only possesses an innate immune system^12^. *Drosophila* innate immunity consists of two pathways: the immune deficiency (Imd) and Toll pathway, both of which induce the production of antibacterial and antifungal peptides, including Drosomycin and Diptericin to fight against bacterial or fungal infection. However, how the immune system responds to the cells of different species, particularly from more phylogenetically distant organisms, such as human cells, is still unknown.

Our finding that A549 cells are visually adjacent to host tissues 3 days post-injection while gradually disappearing over time suggested a possibility that the *Drosophila* innate immunity may come into play in rejecting the human tumor cells. To test this possibility, we utilized the Imd pathway component mutants *PGRP-LC*^*Δ5*^, *Dredd*^*D44*^ and *Rel*^*E20*^ for which viable homozygous lines are readily available and widely used. After transplantation of A549 cells into these mutant flies, we examined the impact of innate immunity on the growth of A549 cells and found that the transplanted A549 cells significantly survived longer in the immunodeficient flies—on average 14–16 days for *PGRP-LC*^*Δ5*^ and *Dredd*^*D44*^ hosts and until death for *Rel*^*E20*^ hosts, compared with only 10 days average in control *w*^*1118*^ flies (Fig. 3a–f). Note that the difference in persistence of the A549 cells fluorescence in *w*^*1118*^ host flies in Fig. 3a, compared with the ones in Fig. 1b, may be due to the number of cells injected (see “Materials and methods”). In addition, the transplanted A549 cells seemed to be more widespread with stronger fluorescence in the immunodeficient flies than in the *w*^*1118*^ flies (Fig. 3a). Similar to the injections in *w*^*1118*^ flies, the location of fluorescence was consistent over time for A549 cells in the immunodeficient fly hosts (Fig. 3a, rows 2–4). These observations together suggest that A549 cells can survive longer in flies without a functional innate immune system.

We next investigated the effects of A549 cells when injected into immunodeficient hosts by measuring the hosts’ lifespan. We observed significantly earlier mortality soon after A549 cell injection (Fig. 3g,h). While we consider the existence of A549 cells to be the main cause of the host fly death, it may also be due to the stress associated with injection. To evaluate this possibility, we measured the lifespan of non-injected and PBS injected flies and compared the findings with the A549 cell-injected flies. As a result, we observed that PBS injections caused a minor, but significant increase in mortality of *PGRP-LC*^*Δ5*^ flies compared to non-injected flies, but not in *Dredd*^*D44*^ (Fig. 3g,h), suggesting that injection stress can cause a minor effect on fly host survival. Given that injections with A549 cells cause significant reduction in survival rate compared to controls, human tumor cells likely detrimentally affect fly hosts. Altogether, our results suggest that transplanted human tumor cells are able to survive and cause negative effects on *Drosophila* hosts, and that the fly host’s immune system is a significant impeding factor for the growth of xenografted cells.

## Discussion

In this study, we performed xenograft experiments using different human tumor cell lines and explored the impacts of transplanted tumor cells in the host flies. We identified two factors that could influence the behavior and survival of the transplanted human tumor cells in *Drosophila*: the incubation temperature and the host immune system. The lower growth rate of human tumor cells in *Drosophila* hosts can be explained by both the optimal temperature and phylogenetic and physiological differences between humans and *Drosophila*. The successes of xenografting in zebrafish at relatively low temperatures (28 °C)^20–22^ showed some promise for transplantation in *Drosophila melanogaster* if incubated at temperatures above 28 °C, or by developing a model in more heat-resistant *Drosophila* species. Indeed, several *Drosophila* species in the American Sonoran Desert such as *Drosophila mojavensis* exhibit the temperature resistance and are capable of mating at temperatures up to the mid-30s °C, with a thermal tolerance of up to around 40 °C^23,24^. Utilizing a more heat-tolerant species of *Drosophila* might increase our likelihood of establishing a viable invertebrate xenograft model.

The other important factor is the difference between the immune system of vertebrates and invertebrates like insects including *Drosophila*. While vertebrate models such as mice and zebrafish have both acquired and innate immunity, *Drosophila* possess only innate immunity^12^. We showed that the loss of innate immunity in Imd mutant flies extended the survival of A549 cells post-injection and further reduced the host lifespan (Fig. 3), indicating the role of innate immunity to repel cells originating from more complex organisms and cancer cells, not only bacteria or fungi. The two innate host defense systems in *Drosophila*, Toll and Imd pathways, are homologous to NF-κB signaling in mammals^25^. In this early attempt, we opted to investigate the role of innate immunity using Imd pathway mutants. The Imd pathway contains components which share homologies with mammalian TNF signaling, and in particular, they are functionally homologous to the mammalian TNFR signaling pathway with the activity of *Drosophila* NF-κB homolog *relish* (*rel*)^26,27^. NF-κB activation in mammals involves the phosphorylation of, and subsequent degradation of the inhibiting IκB protein^28^. In *Drosophila*, Rel contains the IκB domain at the C-terminal, and requires cleavage by the caspase Dredd to split Rel into Rel-49 and Rel-68^29–31^. Ultimately the cleaved Rel-68 translocates into the nucleus and binds to and activates the promoters of the antimicrobial peptide genes, similar to the activated NF-κB^27^. The mammalian TNFR signaling also controls transcription of several mammalian antimicrobial peptides (AMPs), including β-defensins, and recent studies have also described the potential utility of AMPs as anticancer agents^32,33^. Similarly, the *Drosophila dlg*-induced tumor is sensitive to the *Drosophila* homologue Defensin, mediated by TNF signaling^34^. With many similarities to TNFR signaling between vertebrates and fly, the *Drosophila* model provides an opportunity to study the activation and responses of the innate immune system exclusively, triggered by the introduction of non-microbial foreign cells. The detailed mechanisms underlying how *Drosophila* innate immunity affects the growth and survival of transplanted human tumor cells will need to be clarified. It will be also interesting to explore the capabilities of *Drosophila* Defensins against mammalian tumor cells.

Our main goal was to take the first step toward the establishment of a new PDX model using invertebrate *Drosophila*. Current xenograft attempts have been conducted only in vertebrate models, such as mice and zebrafish, due to their evolutionarily conserved similarities to the human body and cellular structures. Compared to the long lifespan of these vertebrate models, *Drosophila* have a relatively short lifespan, which makes it simpler to establish the experimental duration and is potentially useful for screening drug safety and toxicity, as we are able to measure the host lifespan after injection. Indeed, with the goal of establishing a “personalized *Drosophila* model”, Bangi et al. developed a pipeline where a colon cancer patient’s genomic profile was analyzed, and the specific mutations were introduced in *Drosophila*^35^. Large-scale drug screening was then conducted in order to select the most effective candidate drug combinations^35^. In contrast, we aim to approach personalized medicine differently, as we have performed direct transplantation of the donor cells that carry the mutation profile of the original donor, not by expressing *Drosophila* orthologs. While we showed that human tumor cells-transplanted flies died earlier than controls, one caveat is that the hosts’ deaths occurred later than the removal of visible fluorescent signals. This leads to a question of whether the death was directly caused by the presence of human tumor cells or due to irreversible damage induced by the transplanted cells. It is also possible that transplanted tumor cells irreversibly influence host tissues via tumor–host interactions, and this effect leads to lethality. Future studies should focus on the detailed mechanisms of tumor–host interactions from transplantation to host death on a time scale.

In summary, this report showcased the first observations of the behavior of mammalian cell lines when transplanted into *Drosophila* and revealed that temperature and host innate immunity influence the compatibility between mammalian donor cells and *Drosophila* hosts. Further studies will be needed to confirm the value of this model as a novel PDX model as well as future drug screening platforms for cancer research.

## Supplementary Information

Supplementary Figure 1.

## Data Availability

The data that support all experimental findings of this study are available within the paper or from the corresponding author E.K. upon request.
