# Supplementary material for: Drosophila innate immunity suppresses the survival of xenografted mammalian tumor cells
Source: Sci Rep. 2023 Jul 30;13:12334. doi: 10.1038/s41598-023-38489-9 (PMC10387472; doi:10.1038/s41598-023-38489-9)
Supplement: Supplementary file 1 — Supplementary Figure 1. [file 41598_2023_38489_MOESM1_ESM.pdf]

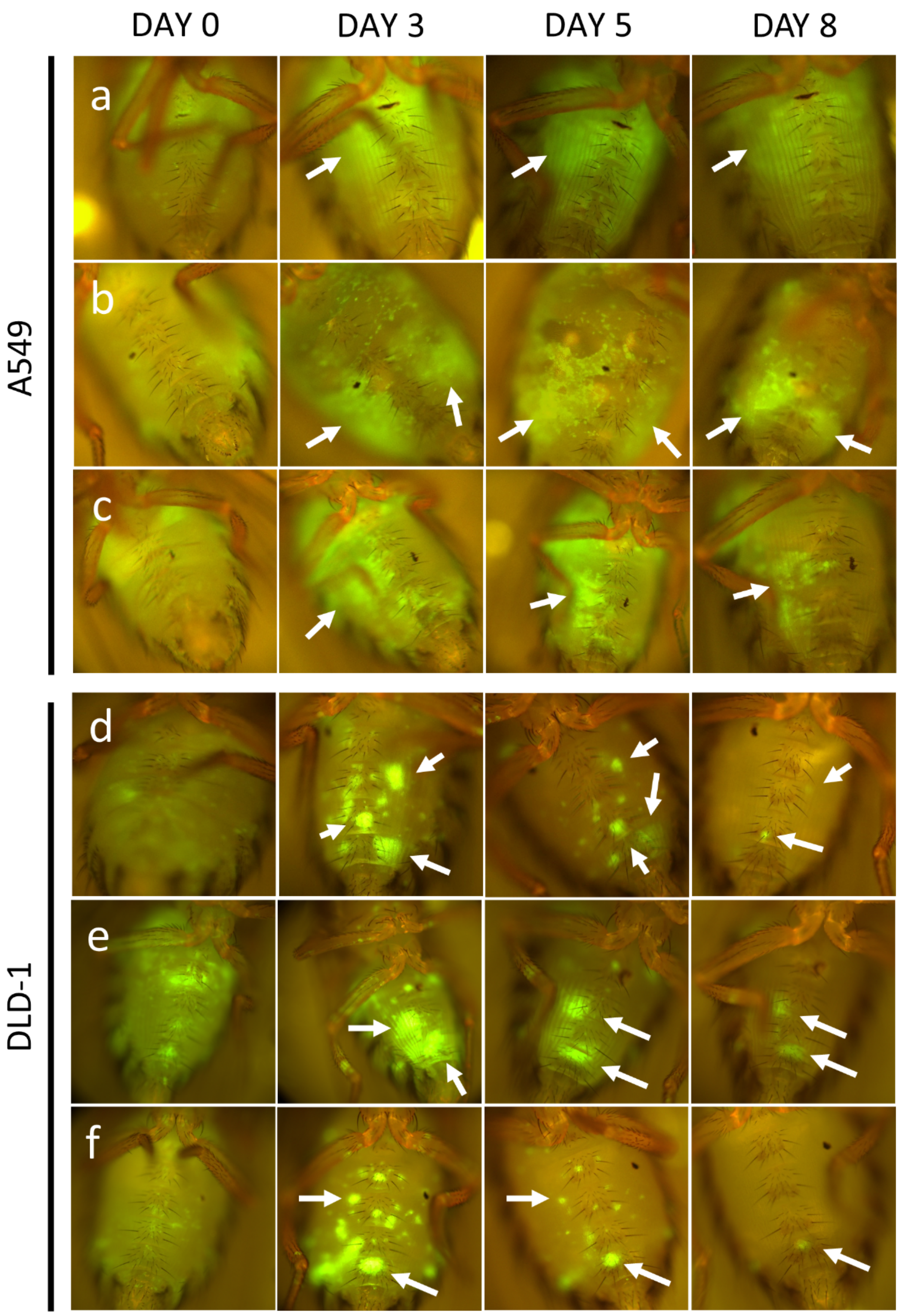


**Supplementary figure 1. A549 and DLD-1 fluorescence patterns in host flies.**

Fluorescence microscopy images of control flies' abdomen injected with A549 (a-c) or DLD-1 (d-f) cells on days 0, 3, 5, 8. Arrows indicate the location of tumors. Cell concentration was $600 \times 10^4$ cells/50 μl PBS. Note the generally spread fluorescence of the A549 cells (n=22/25 spreading), and the clustered DLD-1 fluorescence (n=11/11 clustered). Genotype of host flies: $w^{1118}$.
